# Four days a week and less on appropriate antiviral combinations provided long-term optimal control over HIV-1 in 92 patients

**DOI:** 10.1186/1471-2334-14-S2-O5

**Published:** 2014-05-23

**Authors:** Jacques Leibowitch, Dominique Mathez, Pierre de Truchis, Damien Ledu, Jean Claude melchior, Guislaine Carcelain, Jacques Izopet, John David, Christian Perronne

**Affiliations:** 1 Raymond Poincaré Hospital, Infectiology Department, Garches, France

## Patients and treatments

92 volunteer patients on optimally suppressive antiviral combinations for 5 months or more consented to step wisely reduce their weekly treatment from 7 to 5 to 4 days/wk, or directly from 7 to 4 d/wk after bi-monthly checks on HIV plasma levels at < 50 copies. Weekly treatment was further reduced to 3, 2, and 1 d/wk for respectively 72, 59 and 12 pts. Antiviral combinations included : one integrase inhibitor-base + 2 or 3 NRTIs, or 1 NNRTI and 1 PI (for 4 d/wk regimens, (Rx); standard triple combinations of 1 PI or 1 NNRTI + 2 NRTIs (for 4, 3 , 2 d/wk Rx); novel quadruple antiviral compositions of 1 NNRTI + 3 NRTIs (for 4, 3 , 2, 1 d/wk Rx).

## Results

Intermittent treatment fully controlled patients’ HIV over 20 592 treatment-weeks. Of the 92 patients at entry, 88, 66 and 51 had sustained 52 weeks with 3, 4 or 5 weekly days off-treatment periods respectively. Lymphocyte surface activation markers or cell-bound HIV DNA levels remained stable or declined further; CD4/CD8 ratios rose to ≥1 in 37% of patients – versus 7% patients before discontinuous treatment.

Viral failures: 13 viral escapes (plasma HIV RNA >50 copies 4 weeks apart; overall failures 3.3 per 100 discontinuous treatment-years) were countered by prompt re-adjustments with 7 day-a-week effective combinations. In retrospect 10 failures were ascribable to physician’s or patients’ errors or blunders: base-drug prescribed at sub (½) optimal daily dosage (3 pts); overlooked archival resistant HIVs from antecedent treatment failures (5 pts); acute erratic observance (2 pts); HIV inadvertently resurged while on a 5 day off (2 pts) or 6 days off-treatment period, all 3 under one quadruple combination, setting the antiviral power limit of the ultra-short treatment modality with that combination at 1.4 failures per 100 proper treatment-years.

## Conclusion

discontinuous maintenance therapy in 92 patients on ARV 4 days a week and less over an average 219 treatment-weeks (median 131) offered 40 to 85 % medicinal cuts.

